# Supplementary figures and images for: The ESCRT protein CHMP5 restricts bone formation by controlling endolysosome-mitochondrion-mediated cell senescence
Source: eLife. 2025 Jul 7;13:RP101984. doi: 10.7554/eLife.101984 (PMC12234009; doi:10.7554/eLife.101984)

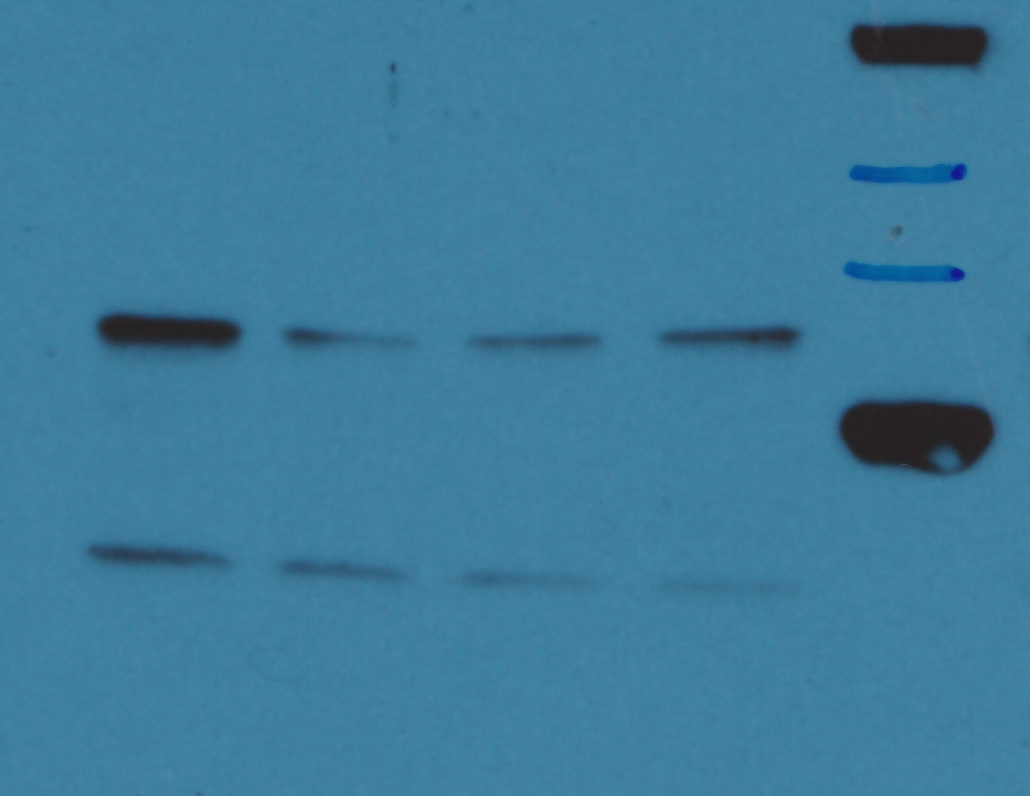

Supplement: Figure 2—source data 2. [file elife-101984-fig2-data2.zip › Figure 2-source data 2/CHMP5.tiff]

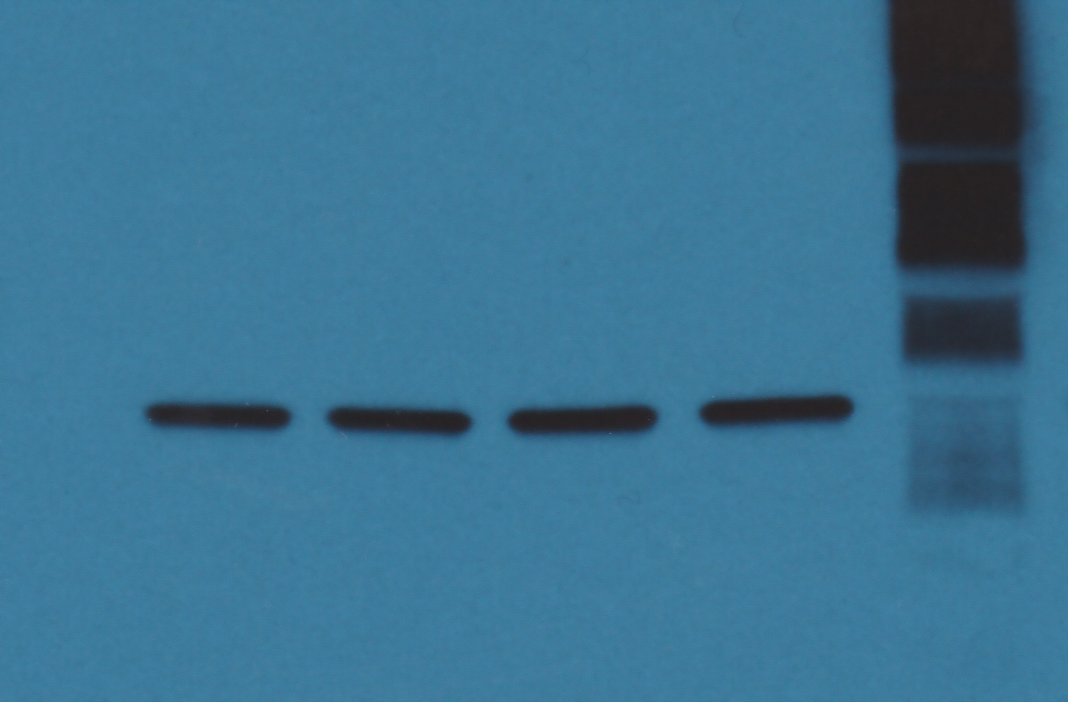

Supplement: Figure 2—source data 2. [file elife-101984-fig2-data2.zip › Figure 2-source data 2/GAPDH.tiff]

CRISPR/CAS9 in MC3T3-E1 cells

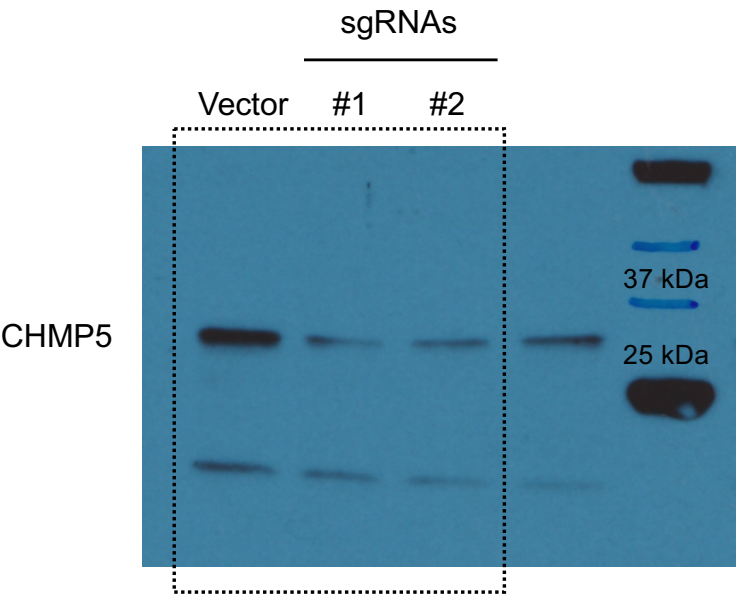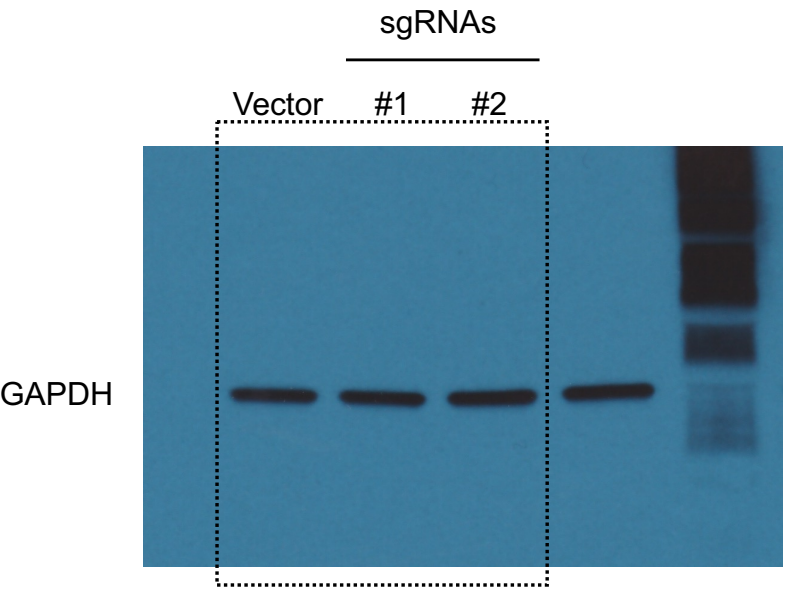

Supplement: Figure 2—source data 3. [file elife-101984-fig2-data3.zip › Figure 2-source data 3.pdf]

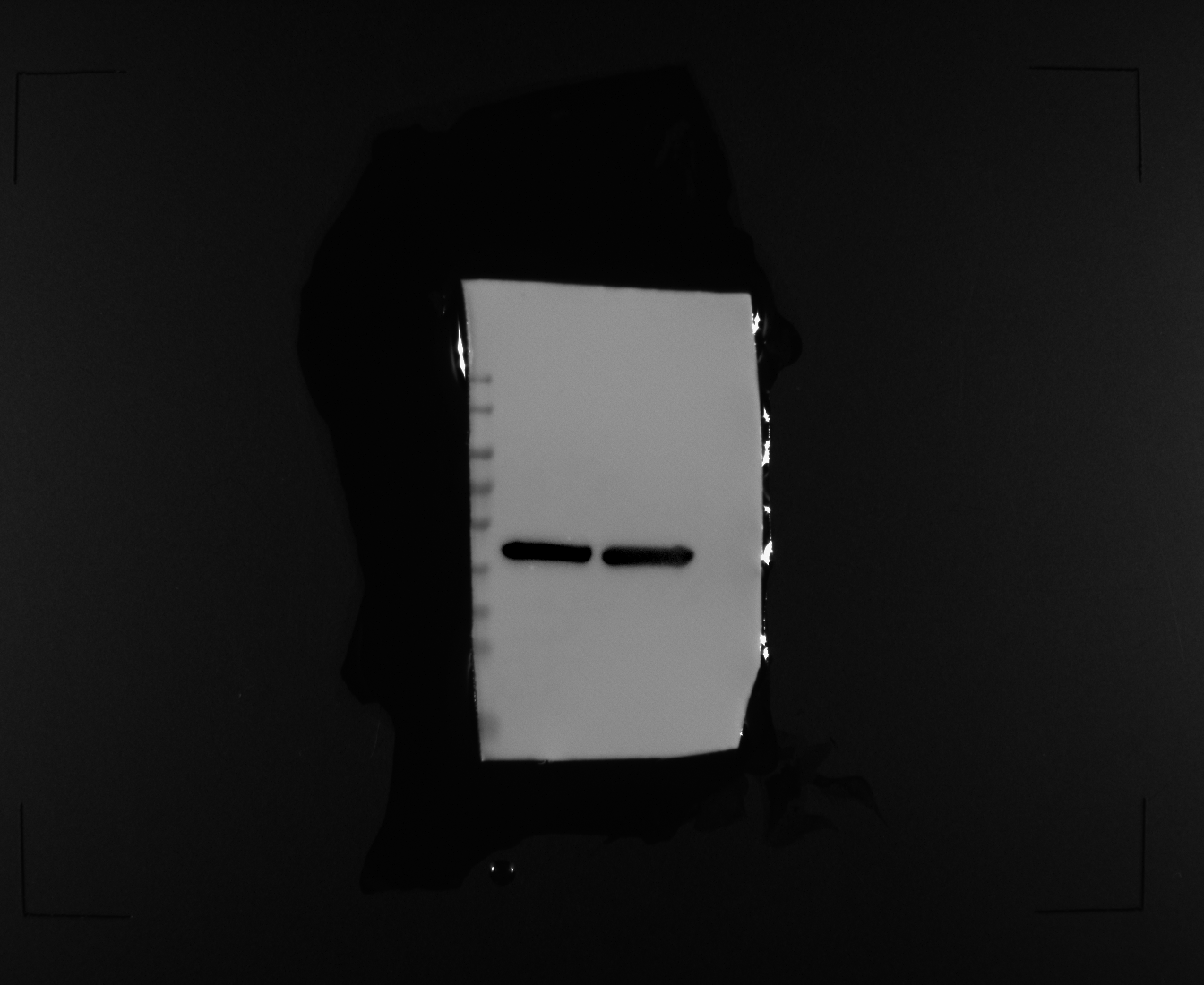

Supplement: Figure 3—source data 2. [file elife-101984-fig3-data2.zip › Figure 3-source data 2/b-Actin for p16.tif]

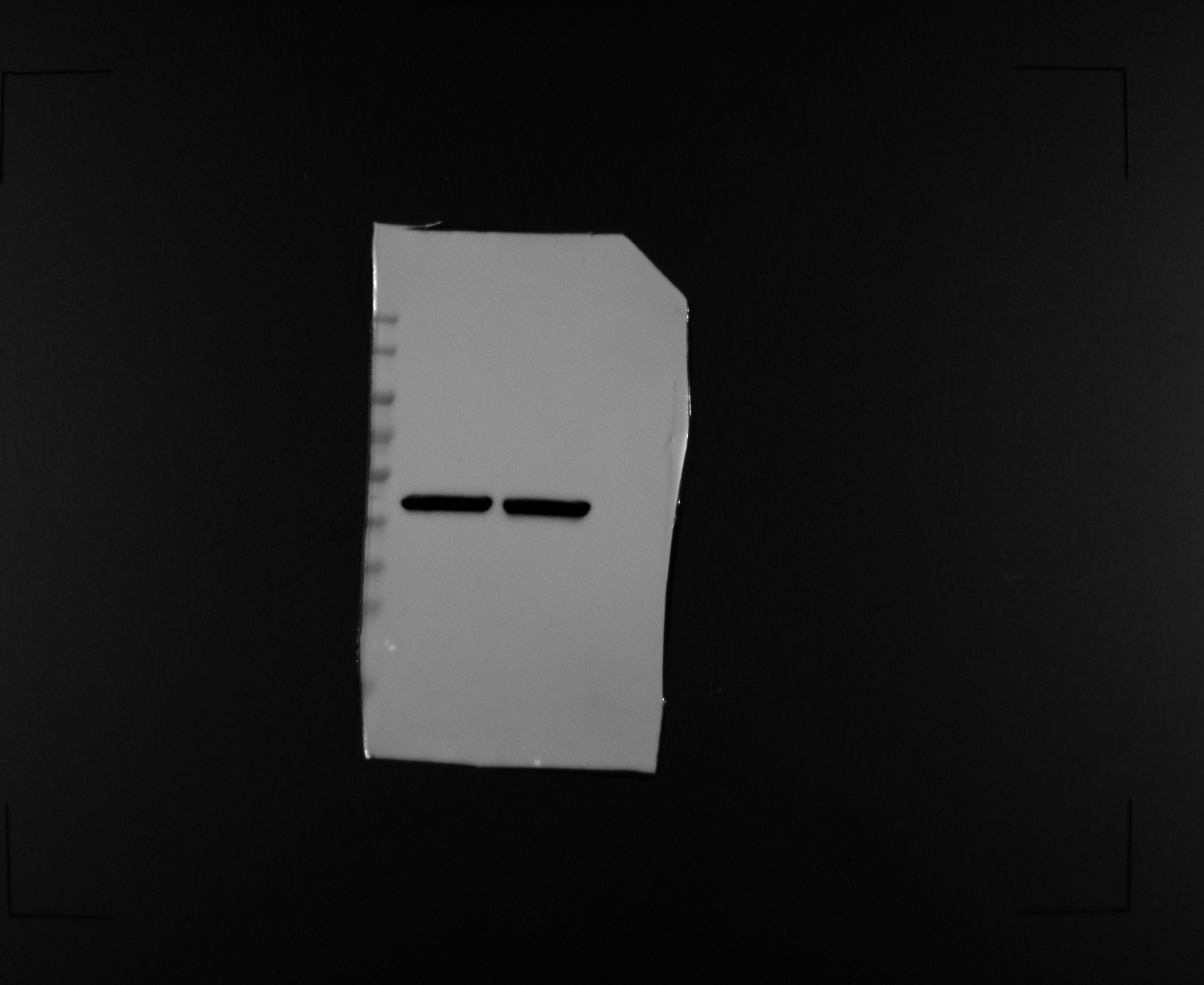

Supplement: Figure 3—source data 2. [file elife-101984-fig3-data2.zip › Figure 3-source data 2/b-Actin for p21.tif]

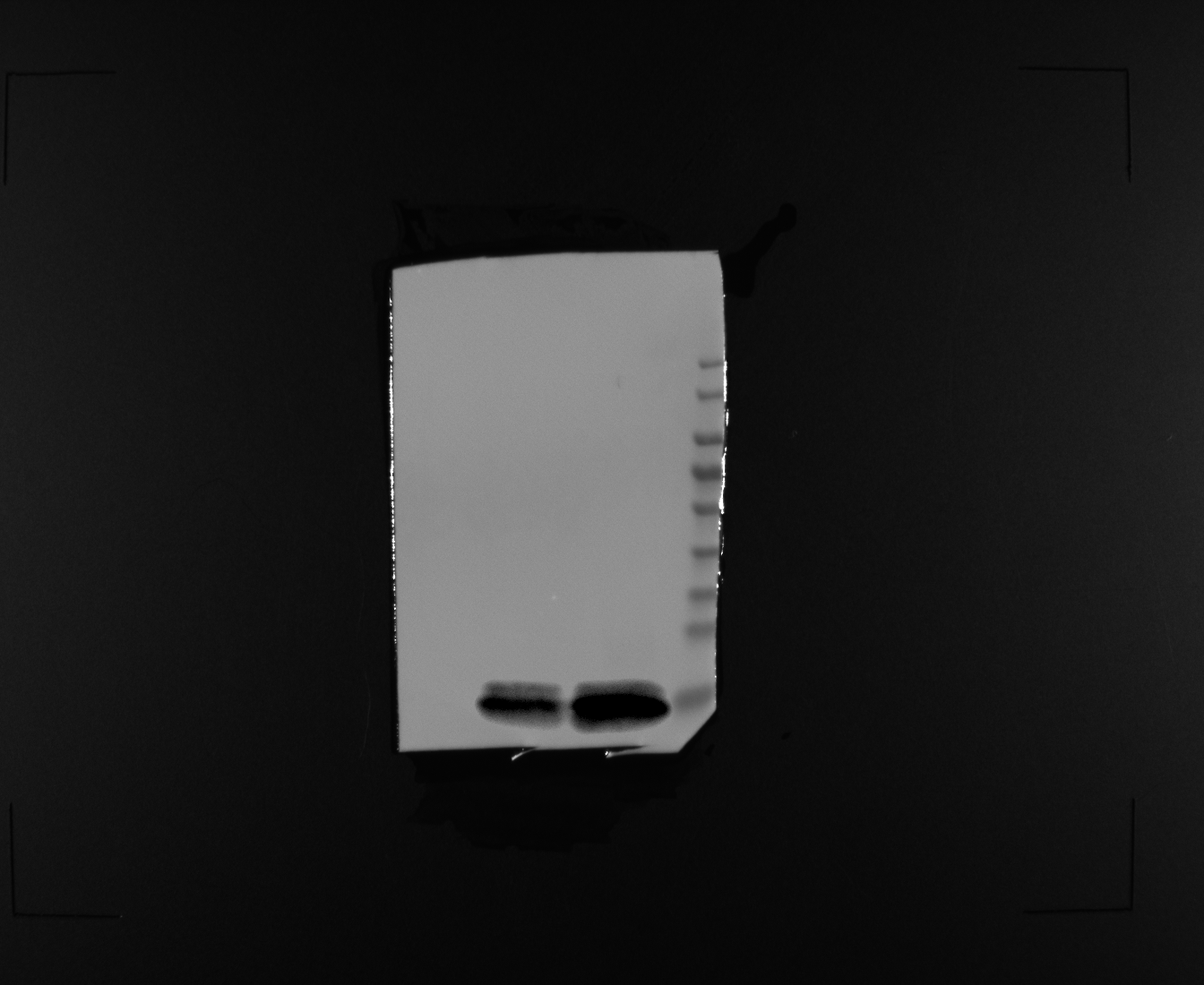

Supplement: Figure 3—source data 2. [file elife-101984-fig3-data2.zip › Figure 3-source data 2/p16.tif]

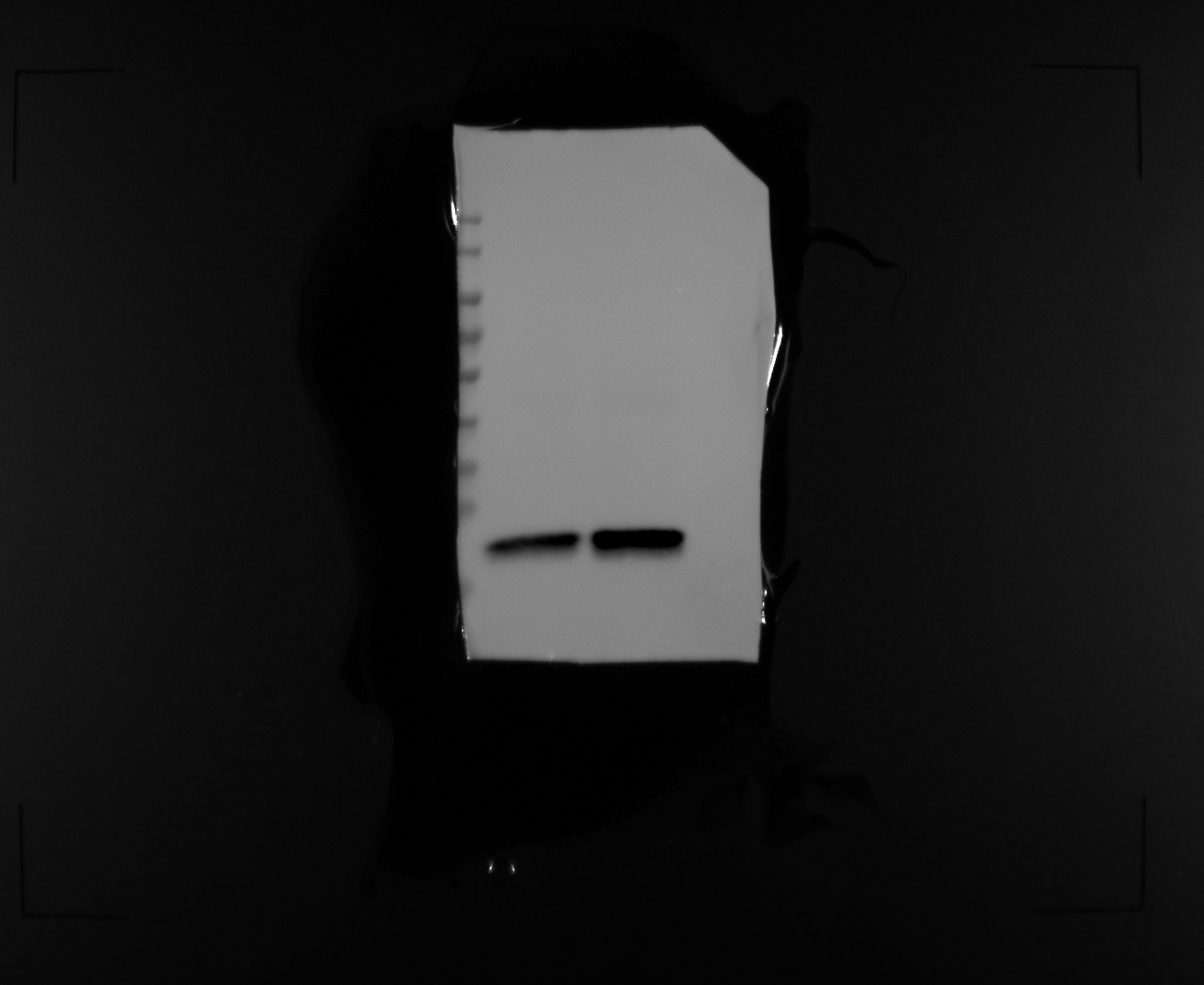

Supplement: Figure 3—source data 2. [file elife-101984-fig3-data2.zip › Figure 3-source data 2/p21.tif]

*Ctsk*<sup>Cre</sup>;  
Control *Chmp5*<sup>fl/fl</sup>

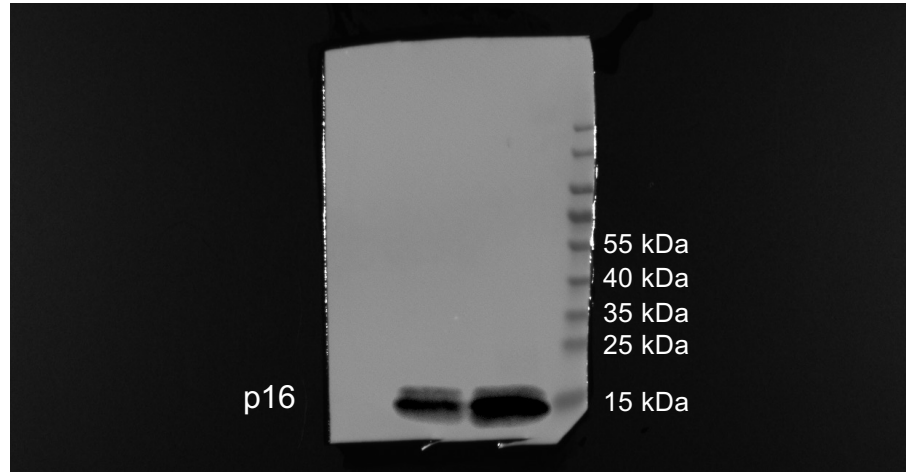

*Ctsk*<sup>Cre</sup>;  
Control *Chmp5*<sup>fl/fl</sup>

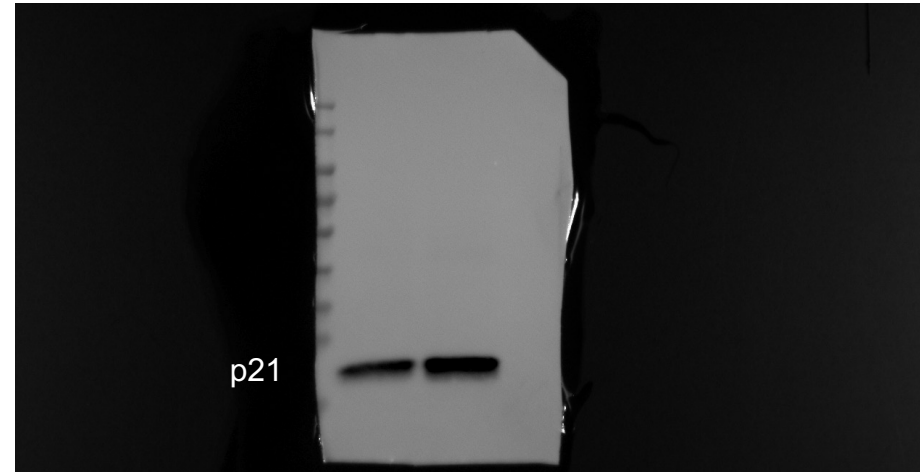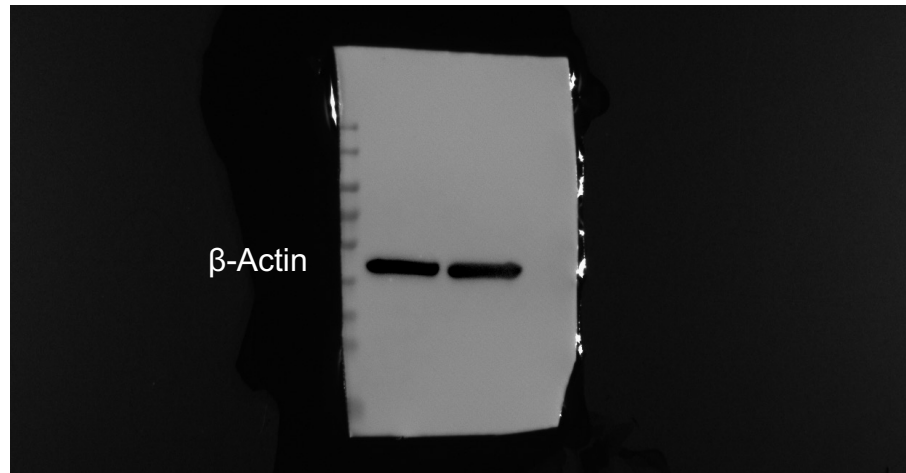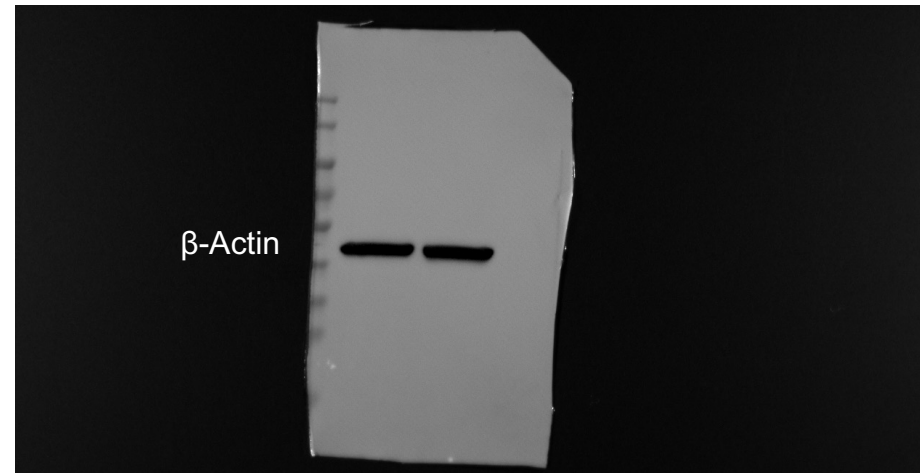

Supplement: Figure 3—source data 3. [file elife-101984-fig3-data3.zip › Figure 3-source data 3.pdf]

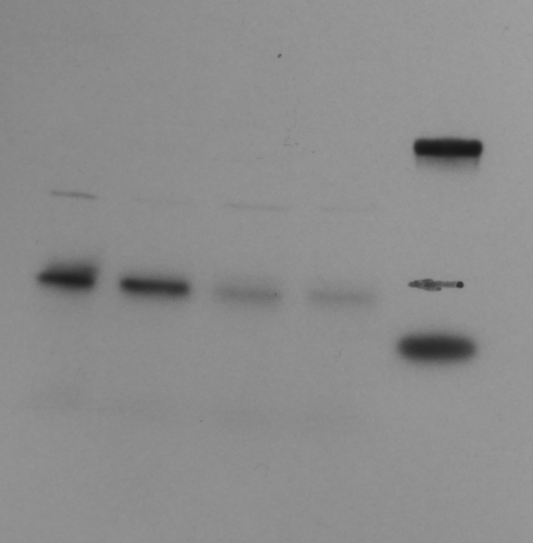

Supplement: Figure 3—figure supplement 1—source data 2. [file elife-101984-fig3-figsupp1-data2.zip › Figure 3-figure supplement 1-source data 2/CHMP5.tif]

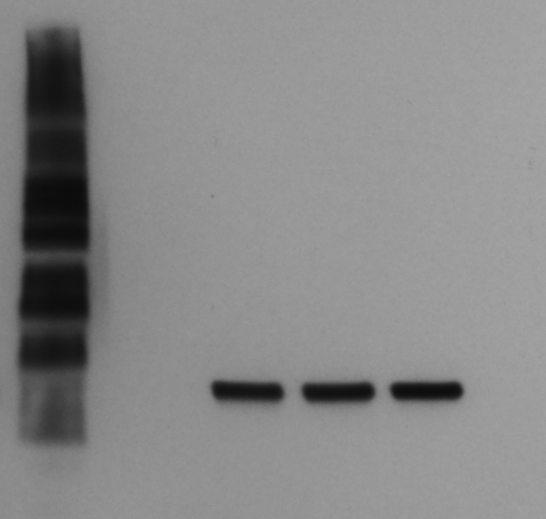

Supplement: Figure 3—figure supplement 1—source data 2. [file elife-101984-fig3-figsupp1-data2.zip › Figure 3-figure supplement 1-source data 2/GAPDH.tif]

# CRISPR/CAS9

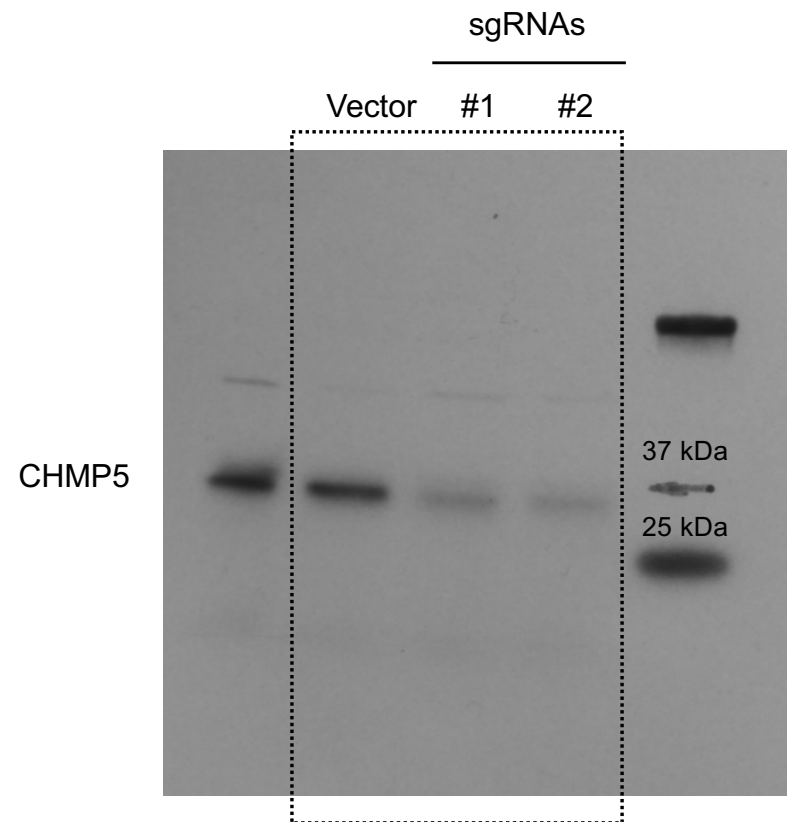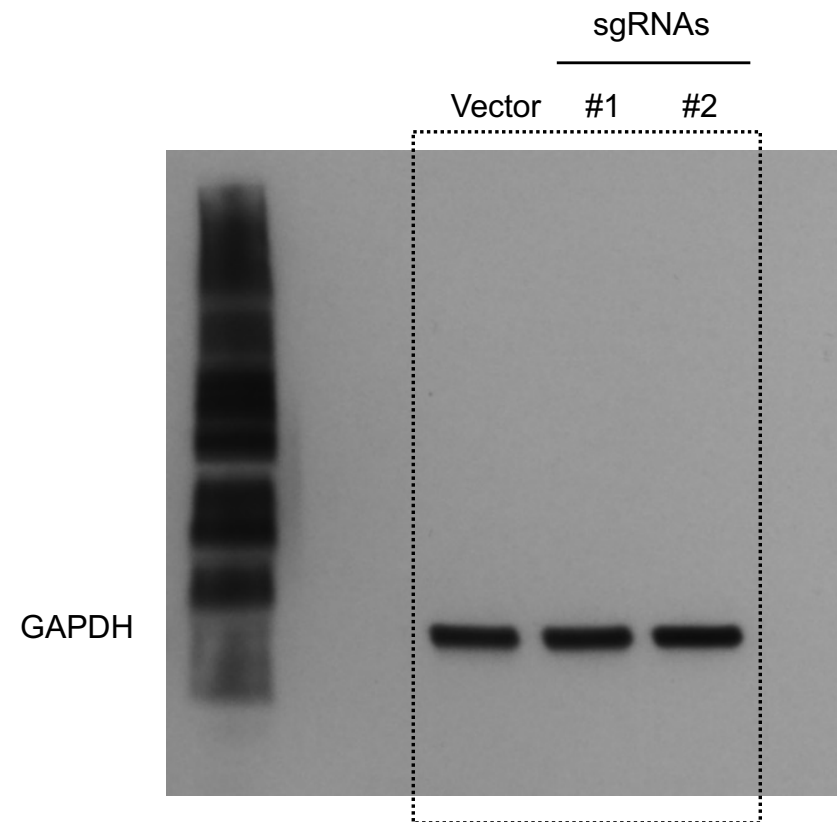

Supplement: Figure 3—figure supplement 1—source data 3. [file elife-101984-fig3-figsupp1-data3.zip › Figure 3-figure supplement 1-source data 3.pdf]

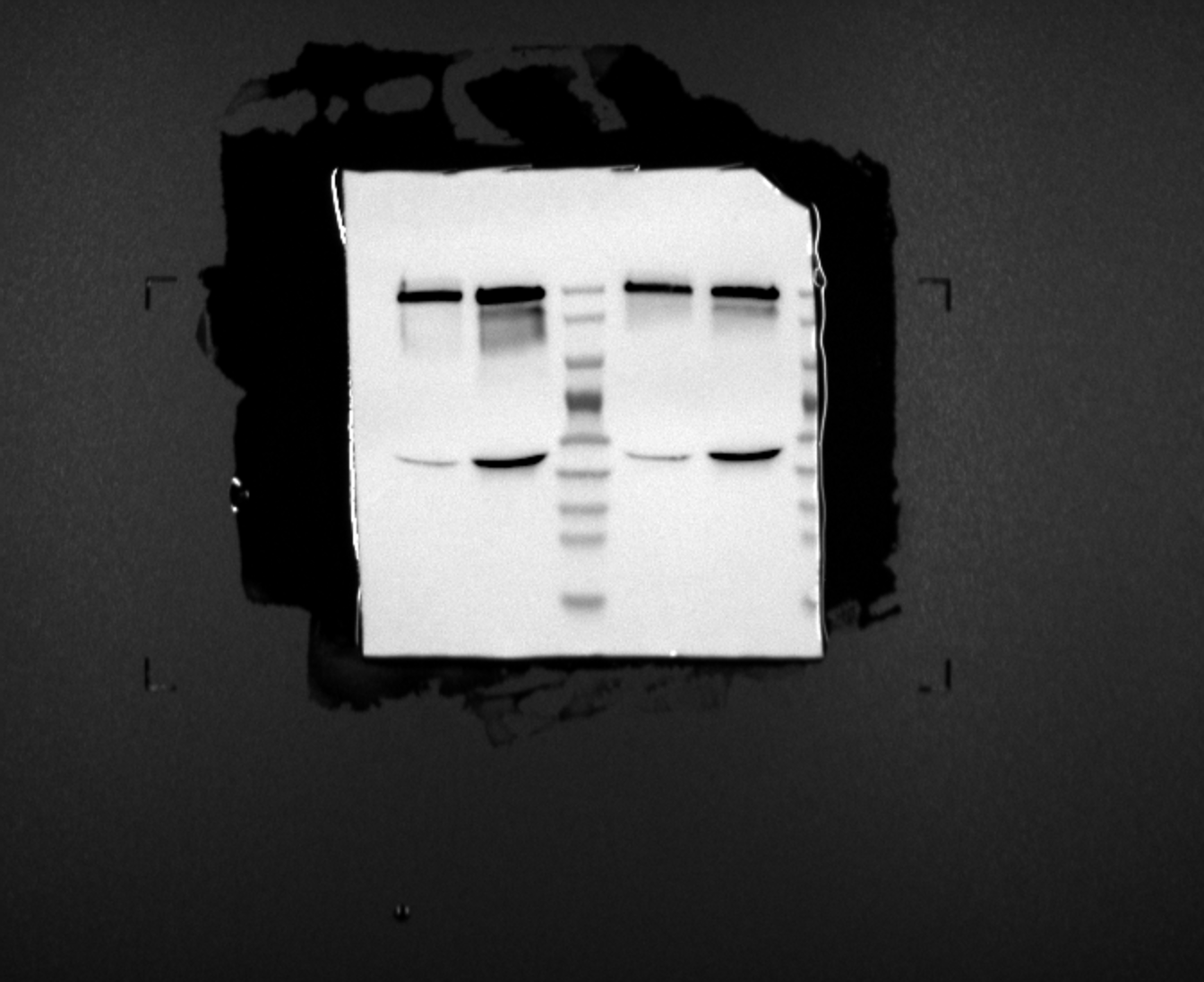

Supplement: Figure 4—source data 2. [file elife-101984-fig4-data2.zip › Figure 4-source data 2/COL1A1.Tif]

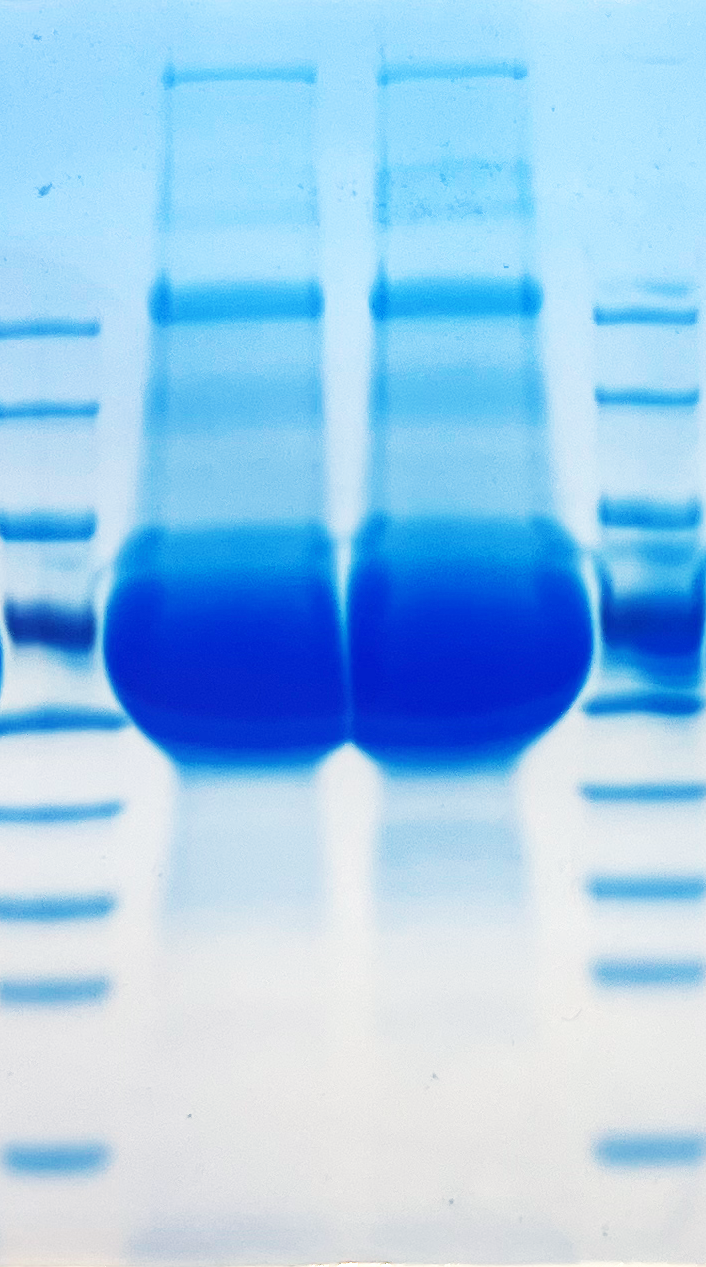

Supplement: Figure 4—source data 2. [file elife-101984-fig4-data2.zip › Figure 4-source data 2/Commas blue.tif]

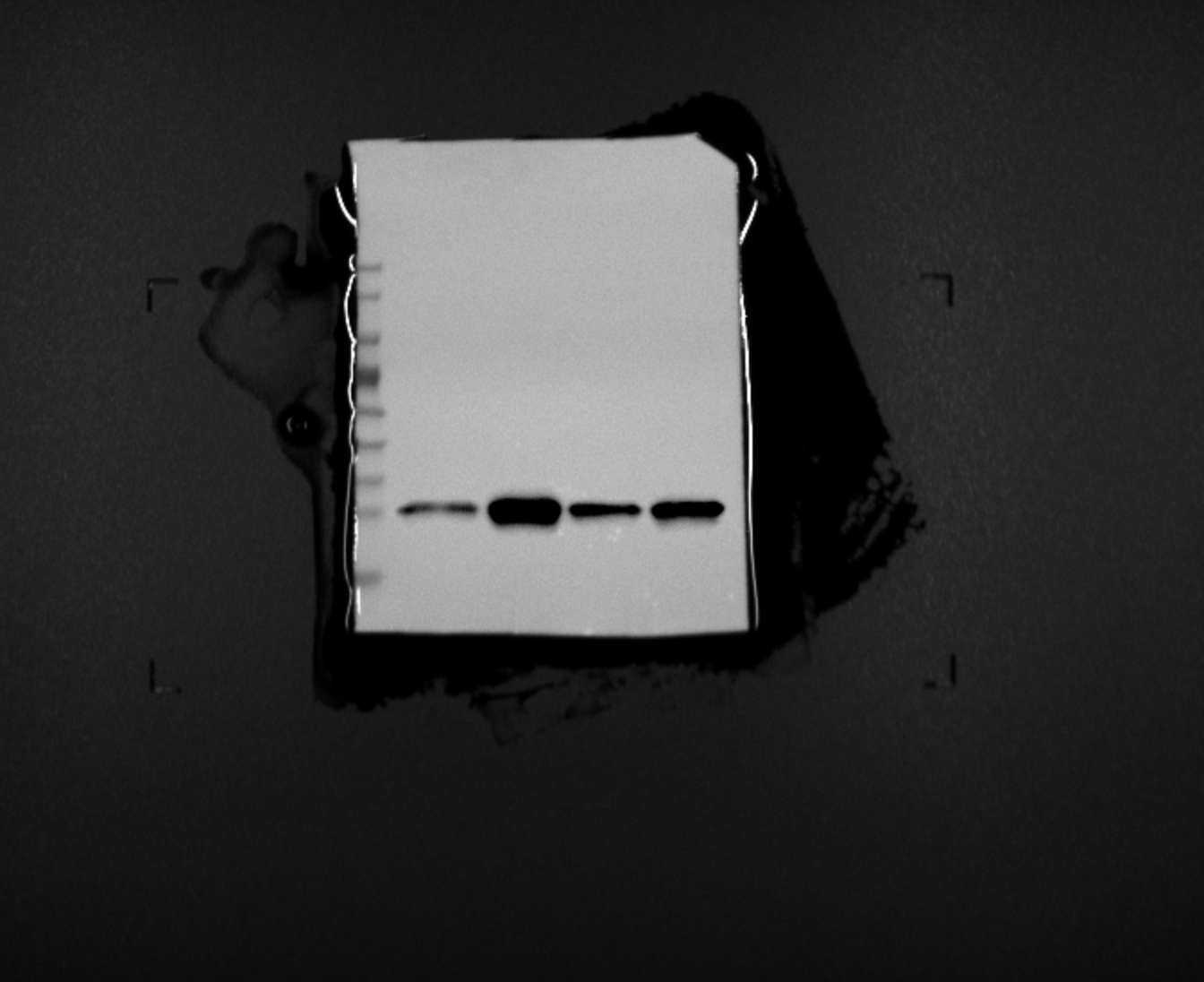

Supplement: Figure 4—source data 2. [file elife-101984-fig4-data2.zip › Figure 4-source data 2/TAGLN.Tif]

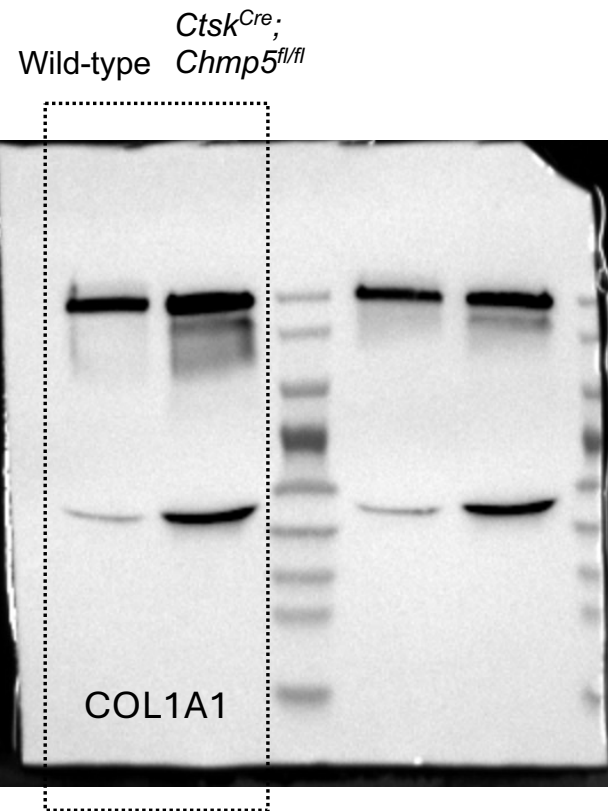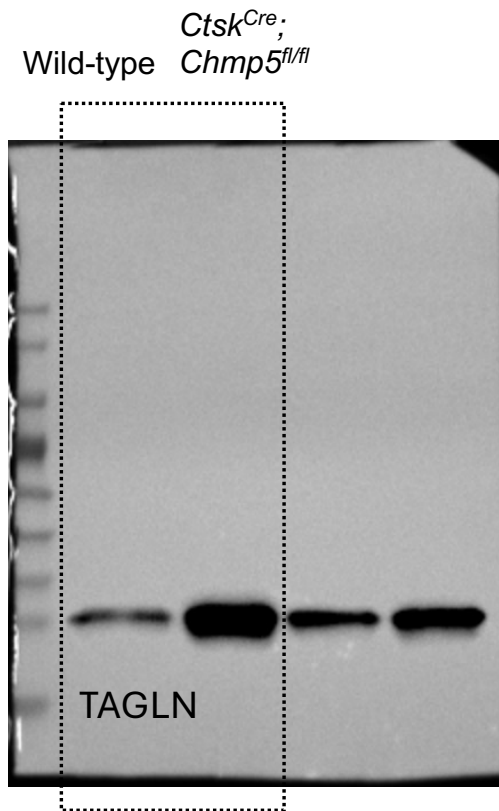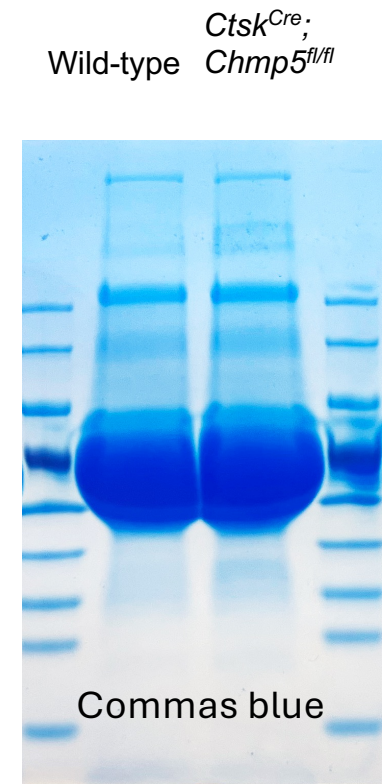

Supplement: Figure 4—source data 3. [file elife-101984-fig4-data3.zip › Figure 4-source data 3.pdf]

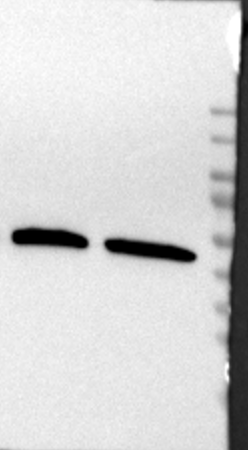

Supplement: Figure 6—source data 2. [file elife-101984-fig6-data2.zip › Figure 6-source data 2/b-Actin.tif]

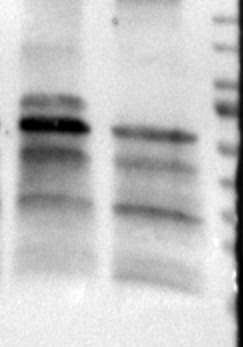

Supplement: Figure 6—source data 2. [file elife-101984-fig6-data2.zip › Figure 6-source data 2/VPS4A.tif]

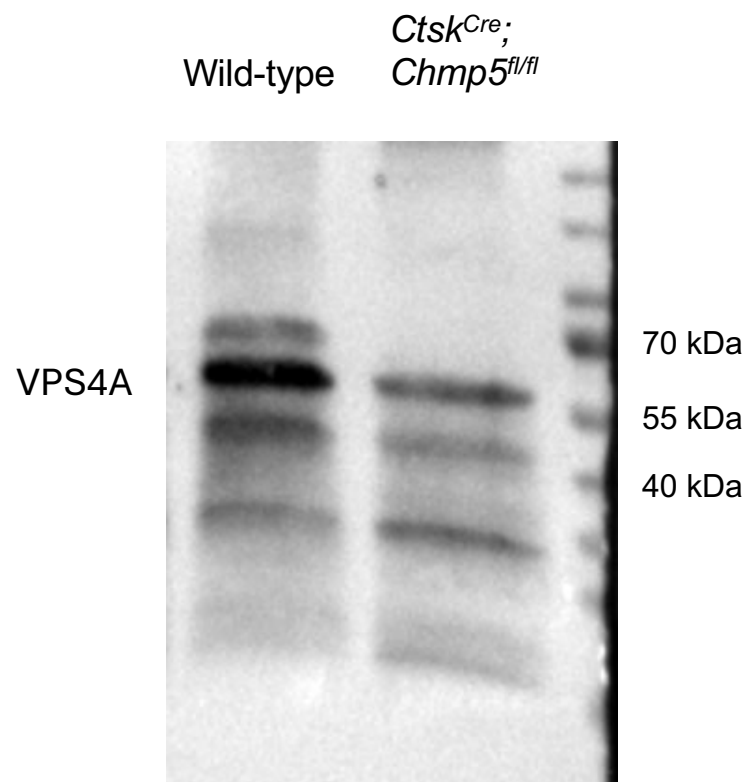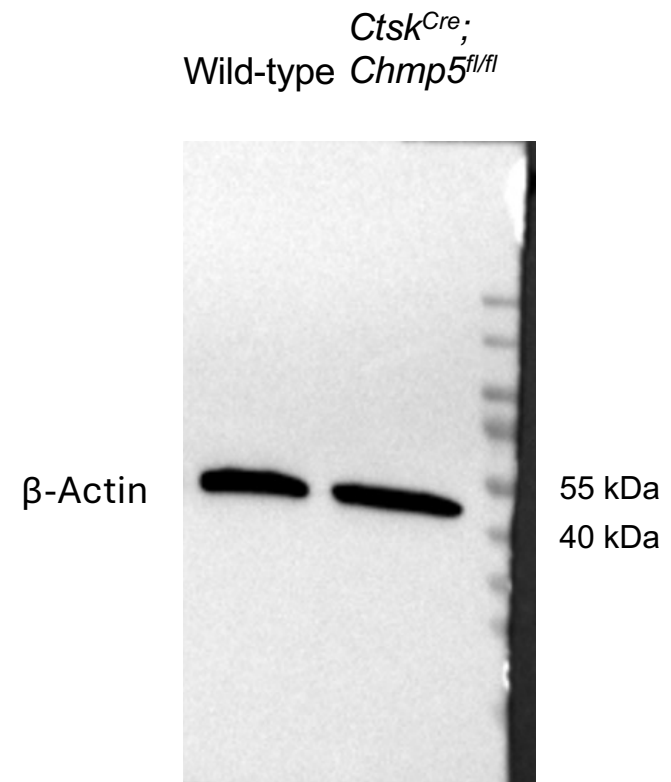

Supplement: Figure 6—source data 3. [file elife-101984-fig6-data3.zip › Figure 6-source data 3.pdf]

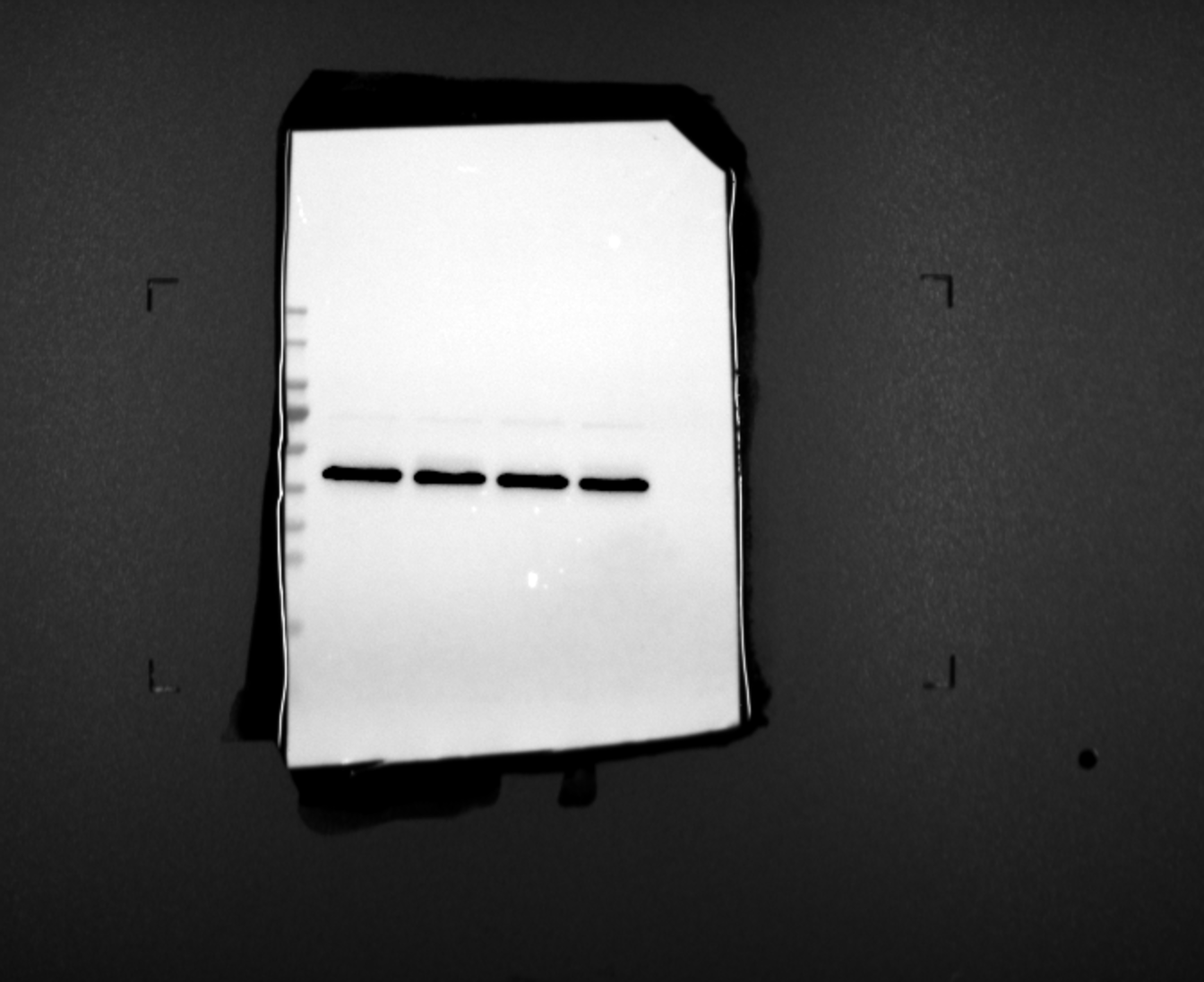

Supplement: Figure 6—figure supplement 1—source data 2. [file elife-101984-fig6-figsupp1-data2.zip › Figure 6-figure supplement 1-source data 2/b-Actin.Tif]

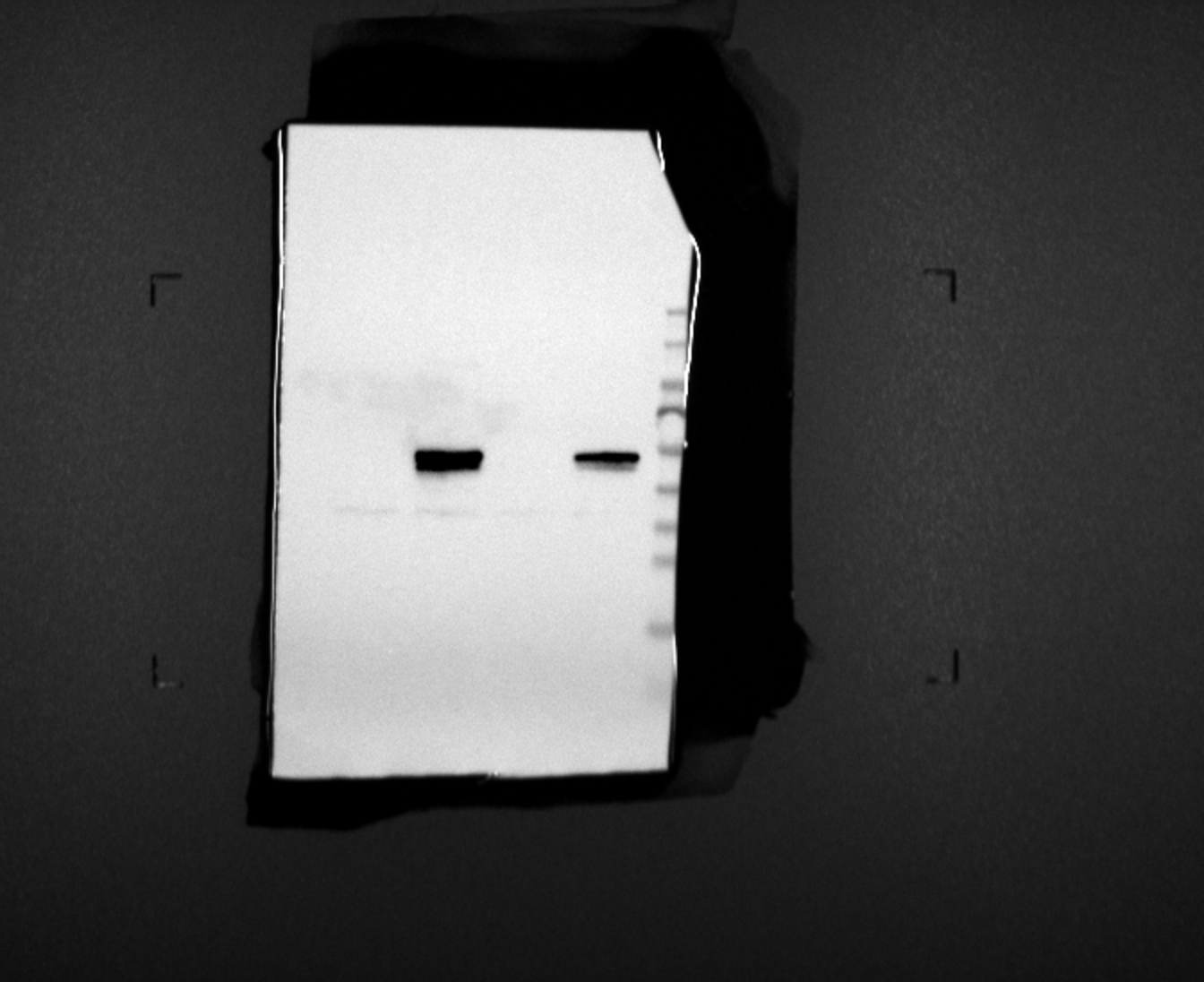

Supplement: Figure 6—figure supplement 1—source data 2. [file elife-101984-fig6-figsupp1-data2.zip › Figure 6-figure supplement 1-source data 2/CHMP5.Tif]

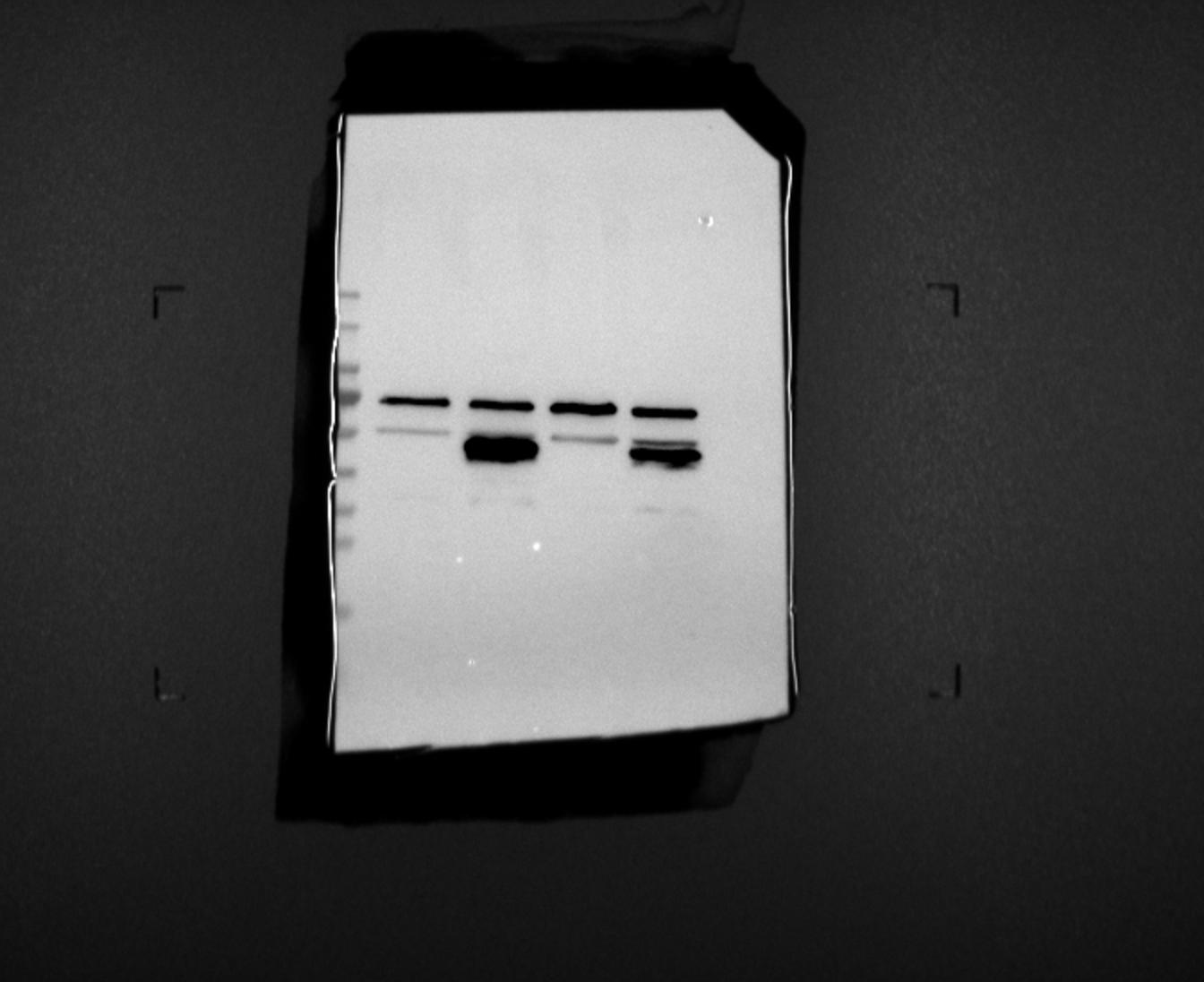

Supplement: Figure 6—figure supplement 1—source data 2. [file elife-101984-fig6-figsupp1-data2.zip › Figure 6-figure supplement 1-source data 2/VPS4A.Tif]

Vector CHMP5-flag

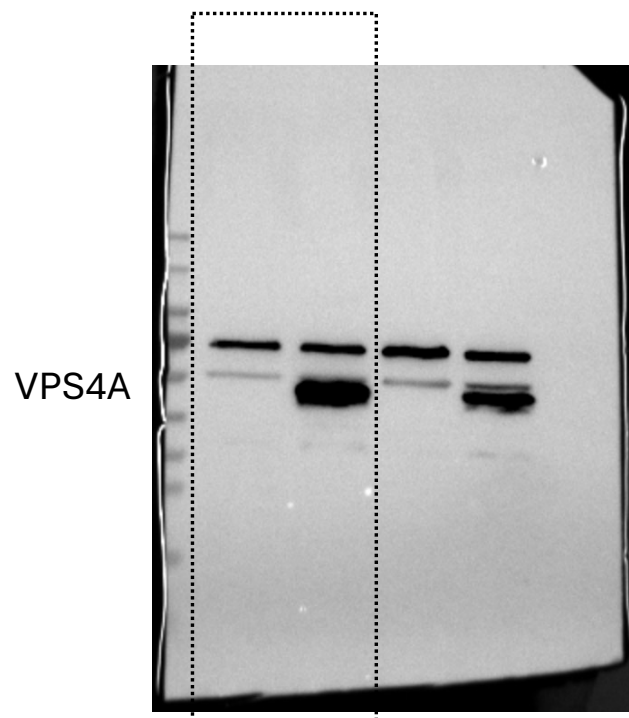

Vector CHMP5-flag

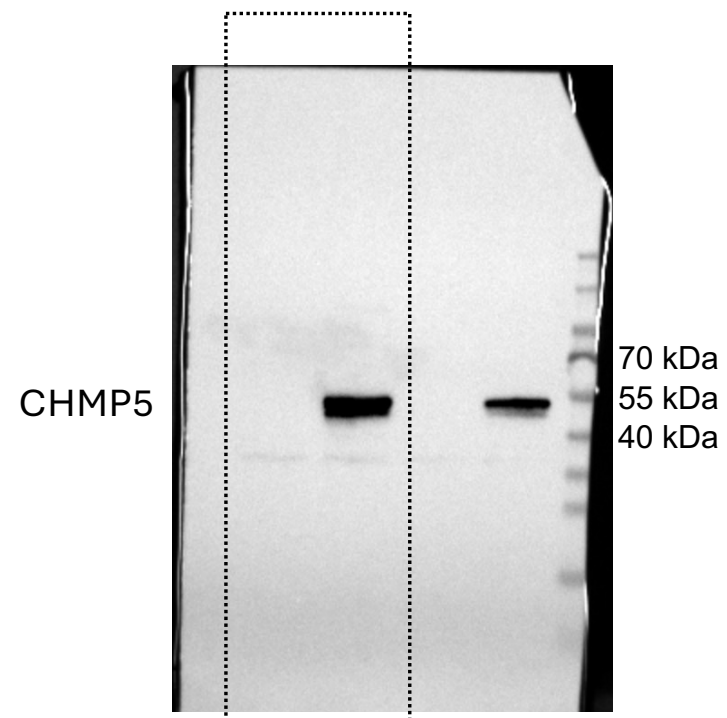

Vector CHMP5-flag

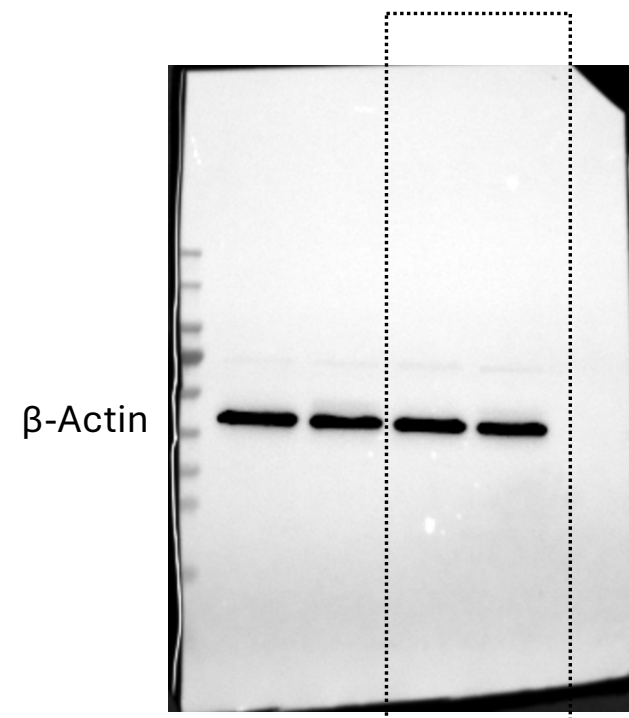

Supplement: Figure 6—figure supplement 1—source data 3. [file elife-101984-fig6-figsupp1-data3.zip › Figure 6-figure supplement 1-source data 3.pdf]

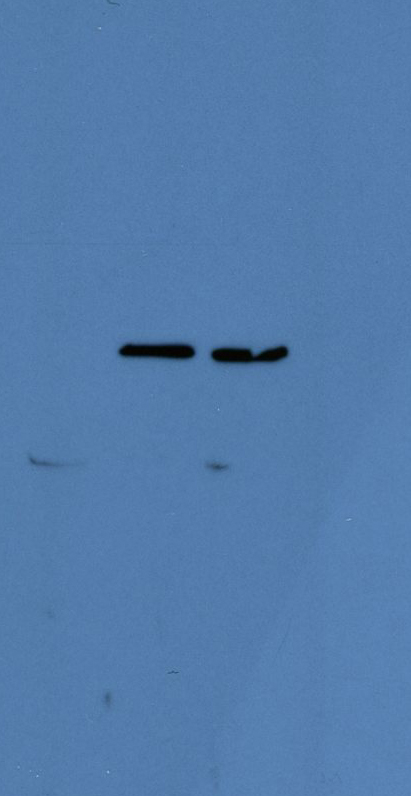

Supplement: Figure 7—source data 2. [file elife-101984-fig7-data2.zip › Figure 7-source data 2/b-Actin.jpg]

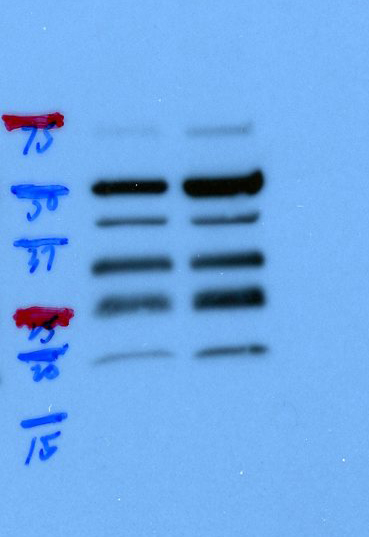

Supplement: Figure 7—source data 2. [file elife-101984-fig7-data2.zip › Figure 7-source data 2/OXPHOS.jpg]
