# Supplementary material for: The ESCRT protein CHMP5 restricts bone formation by controlling endolysosome-mitochondrion-mediated cell senescence
Source: eLife. 2025 Jul 7;13:RP101984. doi: 10.7554/eLife.101984 (PMC12234009; doi:10.7554/eLife.101984)
Supplement: Figure 7—source data 3. [file elife-101984-fig7-data3.zip › Figure 7-source data 3.pdf]

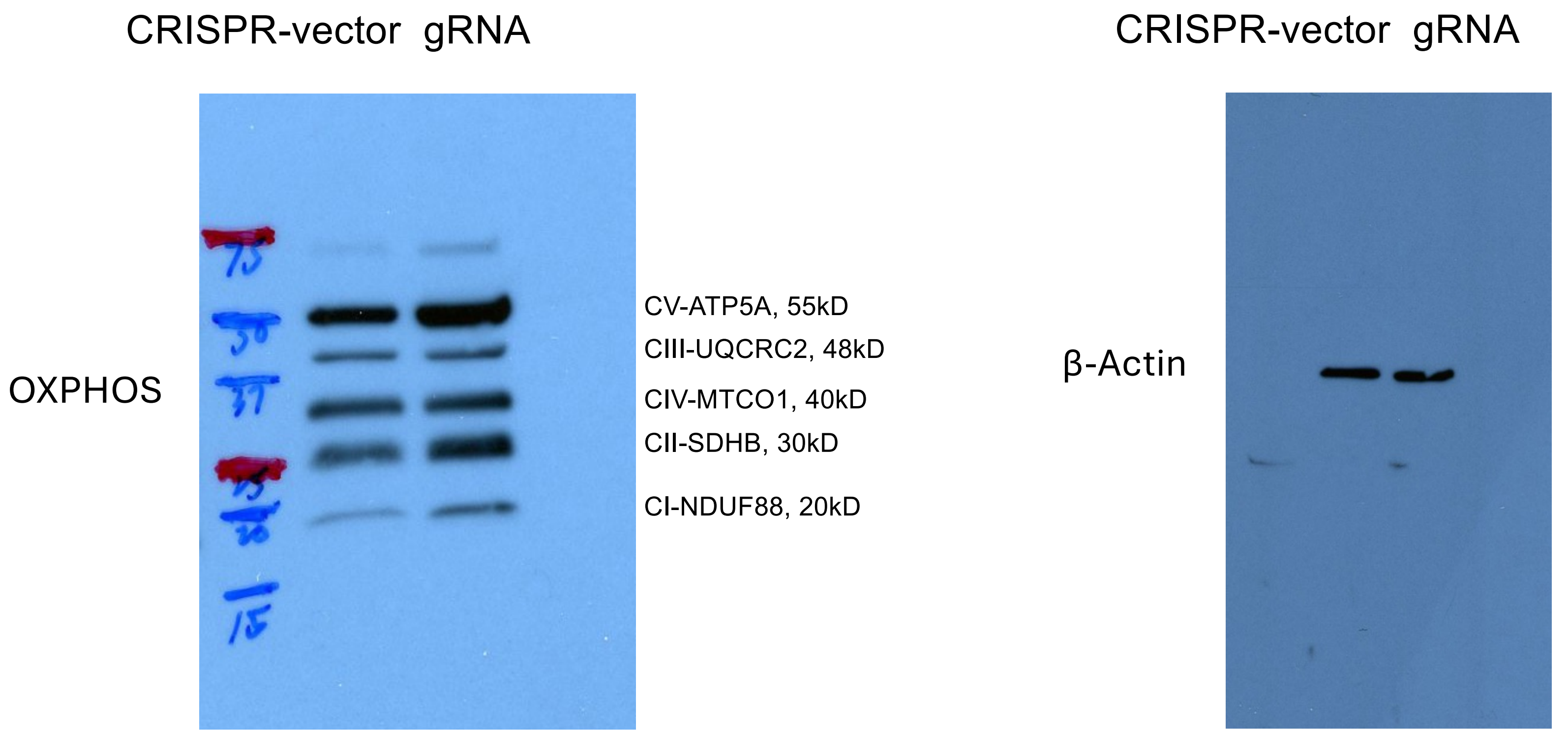
CRISPR-vector gRNA
OXPHOS
CV-ATP5A, 55kD
CIII-UQCRC2, 48kD
CIV-MTCO1, 40kD
CII-SDHB, 30kD
CI-NDUF88, 20kD
CRISPR-vector gRNA
β-Actin
